# Evaluation and classification of right ventricular wall motion abnormalities in healthy subjects by 3-tesla cardiovascular magnetic resonance imaging

**DOI:** 10.1007/s12471-014-0620-2

**Published:** 2014-10-23

**Authors:** S. Quick, U. Speiser, K. Kury, S. Schoen, K. Ibrahim, R. Strasser

**Affiliations:** 1 Department of Internal Medicine and Cardiology, Technische Universitaet Dresden Heart Center University Hospital, Fetscherstr 76, 01307 Dresden, Germany; 2 Klinikum Pirna Division of Cardiology Vascular Medicine, Pneumonology and Intensive Care Medicine, Struppener Straße 13, 01796 Pirna, Germany

**Keywords:** Right ventricular wall motion, CMRI, Wall motion abnormalities

## Abstract

**Background:**

Right ventricular (RV) shape and function serves as an indicator in several types of heart disease such as arrhythmogenic right ventricular dysplasia (ARVD). However, there is no in-depth knowledge of RV motion, even in healthy subjects. The aim of our study was to provide a quantitative analysis of normal variations in RV wall motion in healthy subjects by cardiac magnetic resonance imaging (CMRI).

**Material and methods:**

The study population consisted of 65 consecutive patients referred for the evaluation of cardiac function by 3 Tesla CMR. Balanced steady-state free-precession images were obtained and areas of disordered RV wall motion were evaluated and classified based on a standardised segmental model for the right ventricle.

**Results:**

In 59 patients (90.8 %) wall motion abnormalities (WMA) of the right ventricle were evident. WMA were predominately detected in the apicolateral segments (72 %) compared with mediolateral (24 %, *P* < 0.001) and inferolateral segments (4 %, *P* < 0.001). Dyskinesia was the most frequent wall motion disorder (62.4 %), followed by hypokinesia (20.8 %) and bulging (16.8 %). The mean WMA diameter in the transverse plane (6.4 ± 1.9 mm) was significantly shorter compared with the diameter in the horizontal long-axis (8.1 ± 3.6 mm, *P* = 0.002) and short-axis plane (10.7 ± 4.6 mm).

**Conclusion:**

WMA of the right ventricle are common. Therefore, one should be aware that these nonpathological wall motion disorders can easily be mistaken for a pathological regional wall motion contraction, particularly in ARVD where to date, clear wall motion criteria are lacking.

## Introduction

Understanding right ventricular (RV) shape and function is particularly important in the diagnosis of intrinsic disease of the right ventricle. Abnormal motion of the right ventricle serves as an indicator in several types of heart disease, such as arrhythmogenic right ventricular dysplasia (ARVD) as well as in pulmonary hypertension and congenital heart disease (1–3). However, there is no in-depth knowledge of RV motion and its correlation to the various diseases.

Imaging of the right ventricle remains challenging because of its fibre orientation pattern, complex anatomy and contraction pattern. Furthermore, there is a tendency to interpret RV motion and shape in a manner similar to our understanding of the left ventricle. While the left ventricle is symmetric and ellipsoidal, the shape of the right ventricle is complex. In contrast to the left ventricle, the right ventricle appears triangular when viewed from the side and crescent shaped when viewed in cross section (4). The 3 mm-thick free wall is thin relative to the thick left ventricular (LV) free wall (5, 6). This relative thinness and complex geometry make abnormalities of the right ventricle less common than pathological conditions involving the left ventricle, and this further contributes to a lack of understanding of the normal and diseased state of the right ventricle.

Cardiovascular magnetic resonance imaging (CMRI) allows accurate and reproducible evaluation of RV and LV function, volumes and ejection fractions (7–9). Multiplane image acquisition makes CMRI superior to other imaging techniques, especially for the examination of the right ventricle. To our knowledge, qualitative description of normal variations in RV morphology and wall motion abnormalities (WMA) are scarce (10). However, careful interpretation of the RV pathologies in function and shape are required as the consequences of misinterpretation are potentially harmful to the patient (11) .

The aim of our study was to provide a quantitative analysis of normal variations in RV wall motion in healthy subjects by CMRI.

## Methods

The study population consisted of 65 consecutive patients (32 women, mean age: 50.6 ± 15.2 years, age range: 21–71 years; 33 men, mean age: 50.6 ± 15.7 years, age range: 22–81 years). Patients were those who were referred to exclude structural heart disease (27.7 %) as part of evaluation of hypertrophic heart disease, ARVD and dilated cardiomyopathy, ischaemic heart disease (55.4 %) and myocarditis (16.9 %). All subjects were shown to be free of heart disease based on the current available guidelines for CMRI (1, 13–16). All the participants provided written informed consent. All the study procedures were in accordance with the ethical standards outlined in the 1975 Declaration of Helsinki, as revised in 1983.

## Cardiovascular magnetic resonance imaging

The 3.0 Tesla magnetic resonance system (Signa HDxt 3.0 T, General Electric’s, Milwaukee, USA) uses an 8-channel cardiac coil and prospective electrocardiographic wave triggering. Real-time scout images in axial, sagittal, and coronal planes were used to localise the cardiac position within the thorax. From ventricular apex to base ECG-triggered, breath-hold, balanced steady-state free precession sequences (SSFP) images were obtained in the short-axis, two-chamber, horizontal long axis (HLA) and transverse view to display cardiac function.

**Fig. 1:**
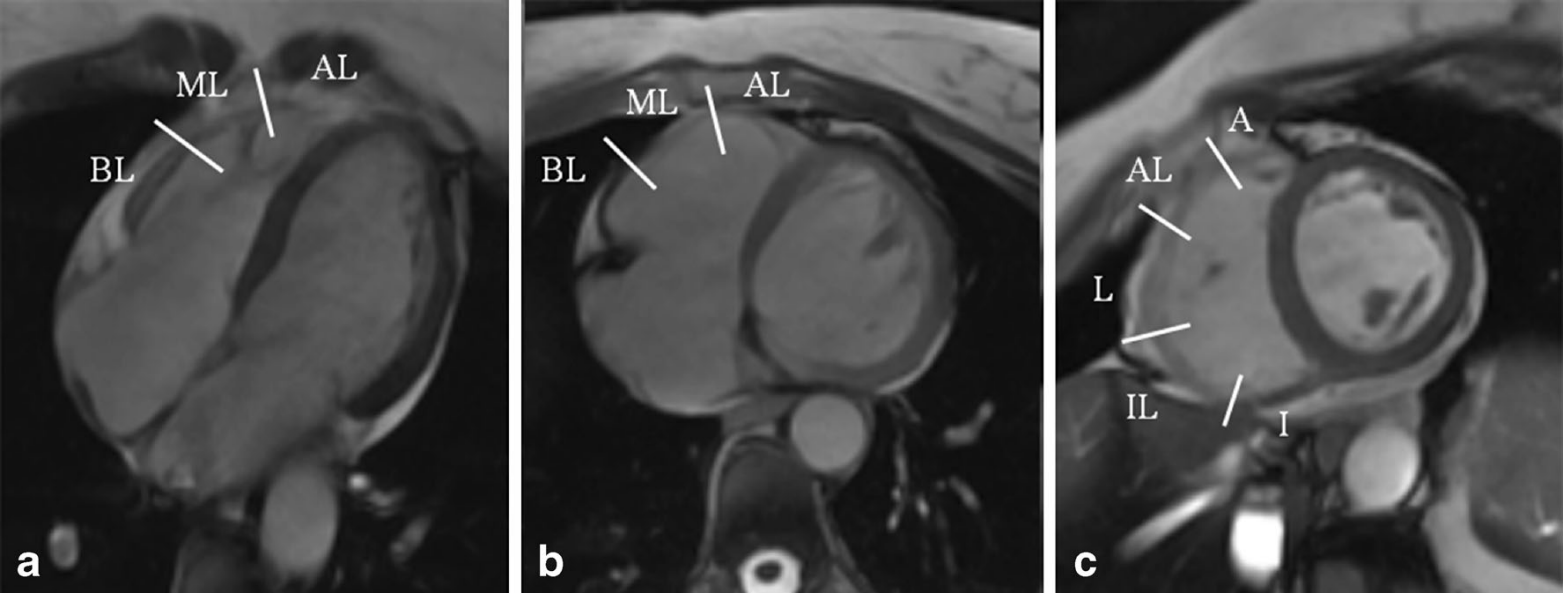
Diagram indicating how the right ventricle can be divided into 11 segments for cardiac magnetic resonance imaging. The longitudinal and short-axis views overlap and complete each other. **a** horizontal long axis, **b** transverse, **c** short axis. BL, Baso-lateral, ML, Medio-lateral, AL, Antero-lateral, I, Inferior, IL, Infero-lateral, L, Lateral, AL, Antero-lateral, A, Anterior

**Fig. 2:**
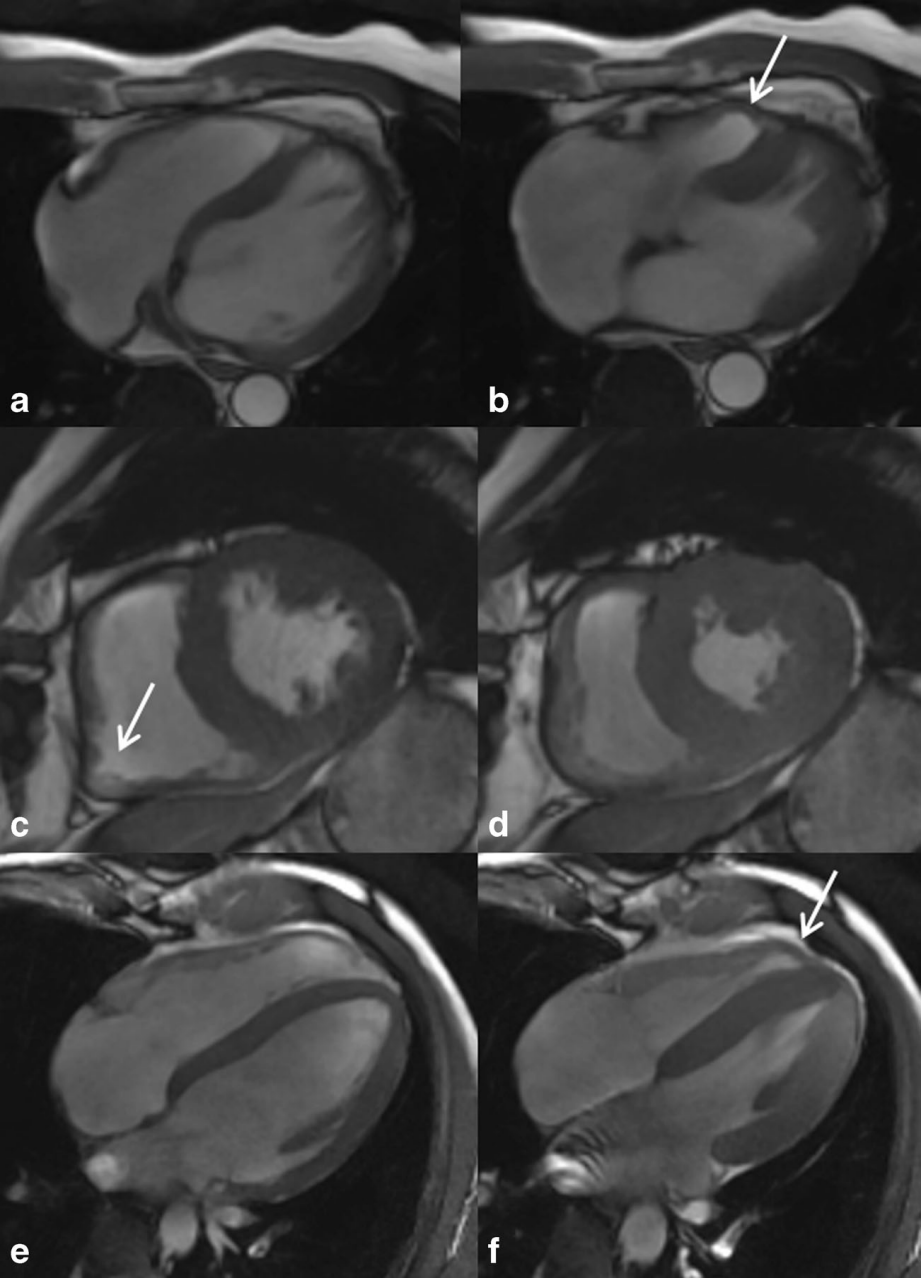
Images of right ventricular wall motion abnormalities in diastole **a, c, e** and systole **b, d, f** acquired in different planes. **a** and **b** Transverse view showing dyskinesia in the mediolateral segment in systole (a, *white arrow*). **c** and **d** Short-axis view with prominent bulging in the inferolateral segment in diastole (c, *white arrow*). **d** and **f** Horizontal long-axis view demonstrating dyskinesia in systole in apicolateral segment (f, *white arrow*)

Parameters were as follows: echo time: 1.6 ms, repetition time =3–6 ms, slice thickness: 8 mm, field of view: 42× 42 cm, read matrix: 256, phase matrix: 256, frames: 26, flip angle: 45°. Ventricular analysis was undertaken by cardiac post-processing software (Report Card 4.0, General Electric’s), which is routinely used for ventricular volume and function analysis. Contraction patterns of the RV free wall were assessed in cine mode in each section plane. Based on a standardised segmental model for the right ventricle, areas of disordered RV wall motion were evaluated and classified (10). The right ventricle was divided into 11 segments, which are evaluated in three planes (short-axis, transverse and HLA plane) in each subject (Fig. 1). WMA were classified as hypokinesia and dyskinesia (in ventricular systole), and bulging (in ventricular diastole) (Fig. 2). In order to further classify the spatial relationship of the affected areas to the moderator band and trabecular muscles, we used the following nomenclature: 1. WMA that are situated between the trabecular muscles, 2. proximal WMA are located mediolaterally, 3. distal WMA are apicolateral and 4. upper WMA are above the insertion of the moderator band. The slices of the various section planes that showed WMA were counted. The size of each WMA was marked in each slice with a cursor and measured by using a conventional software program.

## Statistical analysis

Statistical analysis was performed using Statistical Package of Social Science version 18 (SPSS Inc., Chicago, IL, USA). Qualitative variables were expressed as counts and percentages, and quantitative variables were expressed as mean ±1 SD. Normal distribution was determined by the Kolmogorov*–*Smirnov test (K–S test). Comparison of numerical variables was performed using the unpaired Student *t*-test. Fisher’s exact test was used to examine the dependency of two categorical variables. To compare two groups, the Mann–Whitney *U*-test was used. To compare more than two groups, the Kruskal-Wallis test was used. All reported probability values are two-tailed, and *P* < 0.05 was considered statistically significant.

## Results

We performed CMRI in 65 consecutive patients (32 women, mean age: 50.6 ± 15.2 years, age range: 21–71; 33 men, mean age: 50.6 ± 15.7 years, age range: 22–81). Baseline characteristics and comorbidities of the patient population are demonstrated in Table 1. The study population was an elderly (50.6 ± 15.3 years) cohort with a history of hypertension (30.8 %), dyslipidaemia (23.1 %), diabetes (4.6 %), and nicotine abuse (6.2 %).

**Table 1:** Frequency of the various wall motion abnormalities in relation to different section planes and insertion of the moderator band

	Dyskinesia *n* = 78, (%)	Hypokinesia *n* = 26, (%)	Bulging *n* = 21, (%)
Plane			
HLA	33 (42.3)	14 (53.8)	10 (47.6)
Transverse	45 (57.7)	12 (46.2)	6 (28.6)
Short axis	0	0	5 (23.8)
Relation to MB/Trabecular m.			
Proximal	37 (47.3)	0	1 (4.7)
Distal	18 (23.3)	11 (42.3)	11 (52.3)
Between branches	12 (15.4)	6 (23.1)	0
Above	11 (14)	9 (34.6)	4 (19)

HLA, horizontal long axis, MB, moderator band, Trabecular m., Trabecular muscles

## LV and RV ejection fraction and dimensions

3 T CMRI showed the RV and LV volumes and ejection fractions to be within the normal range in all the subjects; left ventricle: end-diastolic volume (EDV) 130.6 ± 34.5 mL, end-systolic volume (ESV) 45.7 ± 13.3 mL, stroke volume (SV) 85.9 ± 22.5 mL, ejection fraction (EF) 65.7 ± 3.1 %; right ventricle: EDV 107.8 ± 33.9 mL, ESV 40.4 ± 12.9 mL, SV 67.4 ± 22.3 mL, EF 62.4 % ±3.6 %).

## Right ventricular wall motion abnormalities

**Fig. 3:**
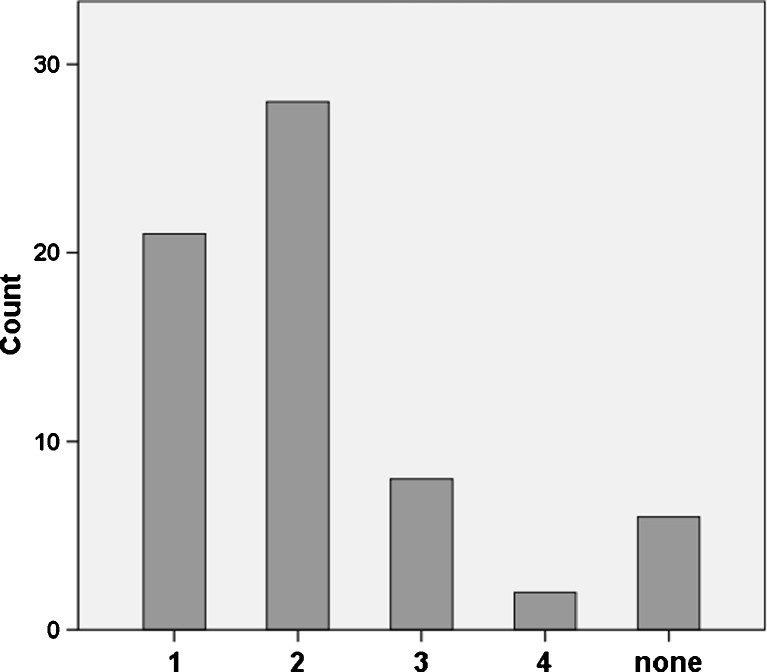
Incidence of healthy subjects according to the number of right ventricular segments affected by wall motion abnormalities

Overall 1275 CMRI slices from 65 patients were analysed for this study. In 59 patients (90.8 %) WMA of the right ventricle were evident. According to the 11 segment model, 6 subjects (9.2) exhibited no RV WMA, in 21 patients (32.2 %) one segment was affected, in 28 (43.1 %) two different segments, in 8 patients (12.3 %) three segments and in 2 subjects (3.1 %) four segments showed WMA (Fig. 3).

**Fig. 4:**
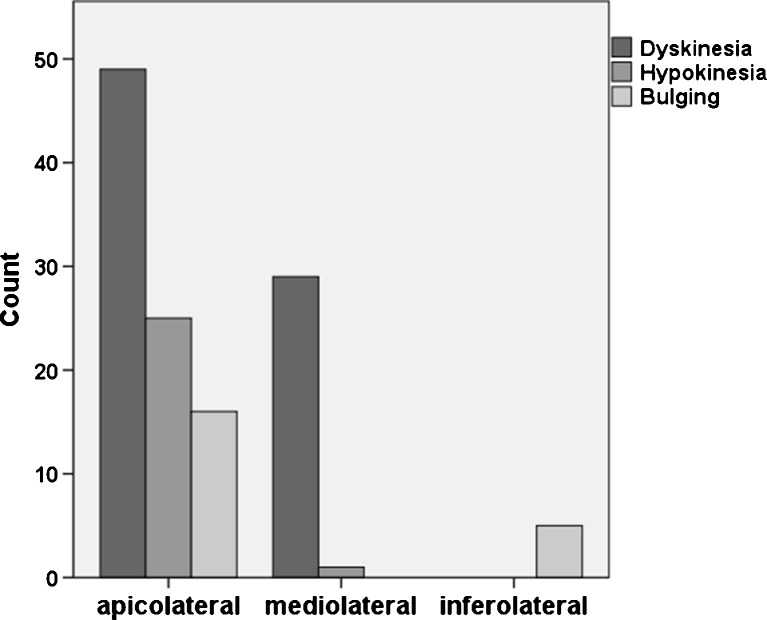
Distribution of wall motion abnormalities. Apicolateral and mediolateral segments of the horizontal long-axis and transverse plane are grouped together

As shown in Fig. 4, WMA were predominately detected in the apicolateral segments (72 %) compared with mediolateral (24 %, *P* < 0.001) and inferolateral segments (4 %, *P* < 0.001).

Dyskinesia was the most frequent WMA (62.4 %), followed by hypokinesia (20.8 %, *P* < 0.001) and bulging (16.8 %, *P* < 0.001). While dyskinesia is predominately seen proximally to the moderator band and trabecular muscle (*P* < 0.001), hypokinetic and bulging segments are more frequently located distally of the moderator band and trabecular muscles (both *P* < 0.001).

As presented in Table 1, WMA of the right ventricle were more frequently seen in the HLA (47.2 %, *P* < 0.001) and the transverse plane (50.4 %, *P* < 0.001) compared with the short-axis plane (2.4 %). Hypokinesia was more often seen in the HLA plane compared with the transverse plane (*P* = 0.02), while dyskinesia was more frequently detected in the transverse rather than the HLA plane (*P* = 0.001).

**Fig. 5:**
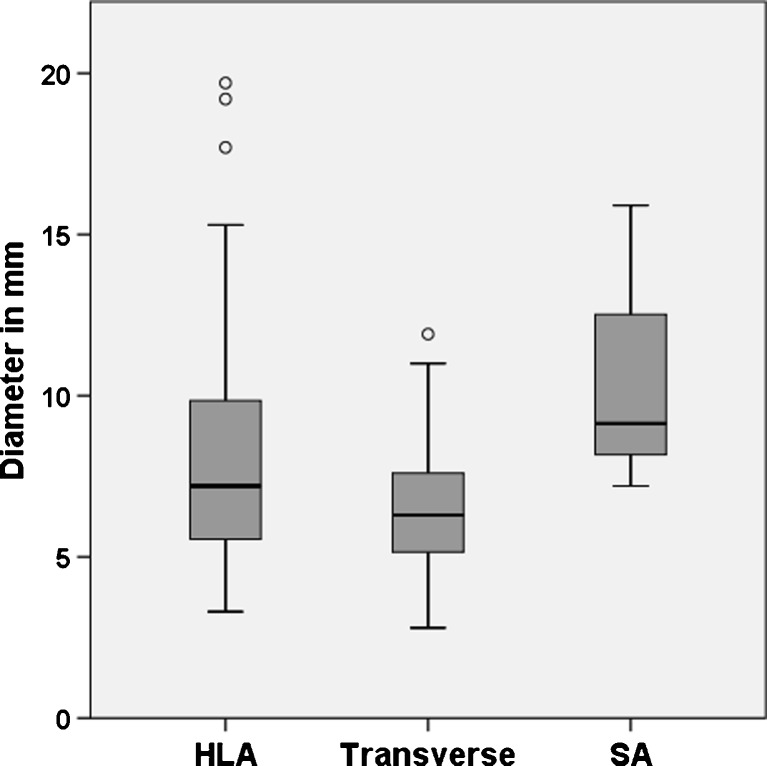
Boxplot of WMA diameters in three different section planes. HLA, horizontal long-axis plane, SA, short-axis plane

Furthermore, differences in the diameter of the WMA in relation to the evaluated segments are obvious. The mean WMA diameter in the transverse plane (6.4 ± 1.9 mm) was significantly shorter compared with the diameter in the HLA (8.1 ± 3.6 mm, *P* = 0.002) and short-axis plane (10.7 ± 4.6 mm, *P* = 0.001) (Fig. 5).

## Discussion

In this study, we have provided a qualitative description of normal variations in RV morphology as observed by CMRI. The main results of this study are the presence of WMA as hypokinesia, dyskinesia and bulging in 92 % of all subjects and the involvement of two or more segments in 60 %. However, the size of the focal WMA was small. No WMA were found in the basal region of the right ventricle; however, most WMA were predominately located in the apicolateral and mediolateral segments with a close spatial relation to the moderator band (79.3 %) and trabecular muscle (19.3 %).

The moderator band or septomarginal trabecula is a constant element of the anatomy of the human right ventricle, characterised by two features: bonding the interventricular septum with the anterior wall of the right ventricle, and its connection with the anterior papillary muscle (17). It could be argued that, because of the close spatial relation, the WMA are due to restraint of the right ventricular free wall by the moderator band (10). On the other hand, the angular variation in RV orientation in the chest may deliver another possible explanation.

Fritz et al. described three major appearances of the right ventricle on images (18). In particular, the box-shaped right ventricle is present in approximately 40 % of normal individuals. This shape is characterised by a nonlinear anterior wall and was frequently associated with focal ‘outpouching’. This focal bulging can easily be mistaken for a pathological regional wall motion contraction.

Finally, the cause of the WMA detected in our healthy cohort will remain a matter of speculation. But the key question is how to differentiate between nonpathological wall motion disorders and pathological WMA to prevent misdiagnosis.

This is an important implication for the CMRI diagnosis of several diseases, in particular for ARVD, in which apparent bulging could be mistaken for pathological WMA or structural defect. The consequences of misinterpretation of the right ventricle by CMRI have been documented (11, 12). The clinical diagnosis of ARVD is complex. Reflecting ARVD studies in general, no ‘gold standard’ for the diagnosis is present. The diagnosis is based on major and minor criteria which take into account structure, function, family history, histology, documented arrhythmia and ECG abnormalities (1). The complexity of diagnosis combined with the potentially devastating consequences of missing the diagnosis frequently precipitates further testing, including CMRI. A definite diagnosis consists of 2 major, or 1 major and 2 minor or 4 minor criteria from different categories. Besides severe dilatation and reduction of the RV ejection fraction, CMRI major diagnostic criteria include localised RV aneurysms (akinetic or dyskinetic areas with diastolic bulging). In the present report 9.3 % of our healthy cohort show dyskinetic areas with concomitant diastolic bulging. Unfortunately, a clear definition, including size and localisation, is not given. Therefore, as outlined above, the ARVD aneurysm criterion on CMRI can be mimicked by either common diseases in uncommon presentations or even by nonpathological wall motion disorders in healthy subjects. A segmental model for the right ventricle, which additionally takes the individual shape of the right ventricle into account, might be helpful for standardisation of the findings and facilitating the comparison of the results of follow-up examinations.

Because it is often very difficult to differentiate normal from pathological CMRI findings, we recommend that interpretation should be performed by well-experienced examiners.

The present study emphasises the advantage of the horizontal long-axis plane. The HLA view is a standardised image plane that is aligned along the long axis of the heart centred at the midventricular level.

In healthy subjects, longitudinal RV shortening is predominant over circumferential RV shortening (19–21). This may be the reason why measurements of the diameter of WMA in the transverse plane are significantly shorter than in the HLA plane. The transverse plane it is not orthogonal relative to the inherent axis of the heart. However, in our study there was no significant difference in the prevalence of the WMA seen in the two views.

Furthermore, the fact that RV contraction is predominantly longitudinal provides an explanation why only bulging is detectable in the short-axis plane. This means that in healthy subjects the HLA plane and the transverse plane clearly show advantages in detecting WMA as dyskinesia and hypokinesia compared with the short-axis plane.

As shown previously, in the heavily mechanically stressed right ventricle, there is a shift from longitudinal to circumferential shortening when compared with the normal right ventricle (22). Consequently, the so-called ‘systemic right ventricle’ contraction pattern resembles that of the normal left ventricle. Therefore, evaluation of WMA of the right ventricle should always carried out and interpreted in regard of the underlying disease.

In conclusion, WMA in the right ventricle are common. Tethering of the free wall by the moderator band or a nonlinear shaped right ventricle are the main reasons for this. In the light of the above, one should be aware that these nonpathological wall motion disorders can easily be mistaken for pathological regional wall motion contraction, particularly in ARVD where to date, clear wall motion criteria are lacking.

## Limitations

Some limitations of the current study should be noted. We acknowledge that this study was based on a small patient cohort. Thus description of normal variations in RV morphology as observed by CMRI are not necessarily definitive and should only be regarded as hypothesis-generating for study in larger patient cohorts.

The subjects included in the study had been referred to our hospital for routine cardiac investigation. All subjects were shown to be free of heart disease based on the current available guidelines for CMRI. However, we cannot exclude that we detected pathologies mimicked by either common diseases in uncommon presentations or rare diseases with typical presentations.
